# An ATR-FTIR Study on the Effect of Molecular Structural Variations on the CO_2_ Absorption Characteristics of Heterocyclic Amines, Part II

**DOI:** 10.1002/cphc.201200066

**Published:** 2012-04-19

**Authors:** Kelly Robinson, Adam McCluskey, Moetaz I Attalla

**Affiliations:** a Coal Technology Portfolio, CSIRO Energy TechnologyNewcastle NSW 2300 (Australia); b Chemistry, School of Environmental & Life Science, The University of NewcastleCallaghan NSW 2308 (Australia)

**Keywords:** absorption, amines, carbon dioxide fixation, IR spectroscopy, nitrogen heterocycles

## Abstract

This paper reports on an ATR-FTIR spectroscopic investigation of the CO_2_ absorption characteristics of a series of heterocyclic diamines: hexahydropyrimidine (HHPY), 2-methyl and 2,2-dimethylhexahydropyrimidine (MHHPY and DMHHPY), hexahydropyridazine (HHPZ), piperazine (PZ) and 2,5- and 2,6-dimethylpiperazine (2,6-DMPZ and 2,5-DMPZ). By using in situ ATR-FTIR the structure–activity relationship of the reaction between heterocyclic diamines and CO_2_ is probed. PZ forms a hydrolysis-resistant carbamate derivative, while HHPY forms a more labile carbamate species with increased susceptibility to hydrolysis, particularly at higher CO_2_ loadings (>0.5 mol CO_2_/mol amine). HHPY exhibits similar reactivity toward CO_2_ to PZ, but with improved aqueous solubility. The α-methyl-substituted MHHPY favours HCO_3_^−^ formation, but MHHPY exhibits comparable CO_2_ absorption capacity to conventional amines MEA and DEA. MHHPY show improved reactivity compared to the conventional α-methyl- substituted primary amine 2-amino-2-methyl-1-propanol. DMHHPY is representative of blended amine systems, and its reactivity highlights the advantages of such systems. HHPZ is relatively unreactive towards CO_2_. The CO_2_ absorption capacity *C*_A_ (mol CO_2_/mol amine) and initial rates of absorption *R*_IA_ (mol CO_2_/mol amine min^−1^) for each reactive diamine are determined: PZ: *C*_A_=0.92, *R*_IA_=0.045; 2,6-DMPZ: *C*_A_=0.86, *R*_IA_=0.025; 2,5-DMPZ: *C*_A_=0.88, *R*_IA_=0.018; HHPY: *C*_A_=0.85, *R*_IA_=0.032; MHHPY: *C*_A_=0.86, *R*_IA_=0.018; DMHHPY: *C*_A_=1.1, *R*_IA_=0.032; and HHPZ: no reaction. Calculations at the B3LYP/6-31+G** and MP2/6-31+G** calculations show that the substitution patterns of the heterocyclic diamines affect carbamate stability, which influences hydrolysis rates.

## 1. Introduction

The dominant sources of anthropogenic CO_2_ emission are fossil-fuel combustion and industrial processes.^[^^1^^,^ ^2^^]^ Sequestration of this CO_2_ is now a major target for reduction of atmospheric CO_2_ levels. While it is clear that any significant long-term reduction in greenhouse gas emissions must involve changing our approach to energy production and consumption, technologies are required to reduce levels in the short term. Coal-fired power stations are the largest point-source emitters of CO_2_ in Australia and worldwide.^[^^3^^]^ The prospect of integrating post-combustion CO_2_ capture (PCC) technology in both existing and new coal-fired power stations offers the potential to lower CO_2_ emissions in the face of existing and predicted growth in the number of coal-fired power stations.^[^^4^^]^

Currently, aqueous amine-based PCC is viewed as the most promising and near-ready technology for the reduction of CO_2_ emissions from coal-fired power stations. PCC involves separating CO_2_ from a flue gas stream by chemical absorption and re-releasing CO_2_ from the absorbent by heating in a two-step process for subsequent storage or industrial use. PCC is industrially proven with absorbents such as aqueous monoethanolamine (MEA), used for decades for CO_2_ removal from gas streams in small-scale commercial processes such as ammonia production and natural-gas processing.^[^^5^^,^ ^6^^]^

Despite being an established technology, deployment of current industry-standard technology (30 wt % aqueous MEA) on a large scale applies a considerable efficiency penalty to the power generation process. Regeneration of PCC absorbent is energy-intensive^[^^7^^]^ and will result in up to 25 % reduction in the net efficiency of a coal-fired power plant.^[^^8^^,^ ^9^^]^ Clearly, the absorption/regeneration characteristics of the amine-based PCC absorbent will influence the economic feasibility of this technology. One approach to reducing the energy requirements and cost of the PCC process is the development of more cost effective and better performing amines. There is considerable scope to develop absorbents that show higher CO_2_ absorption, lower regeneration costs and greater chemical stability, particularly in the face of an increasing move towards demonstration-scale PCC plants.

**Figure 1:**
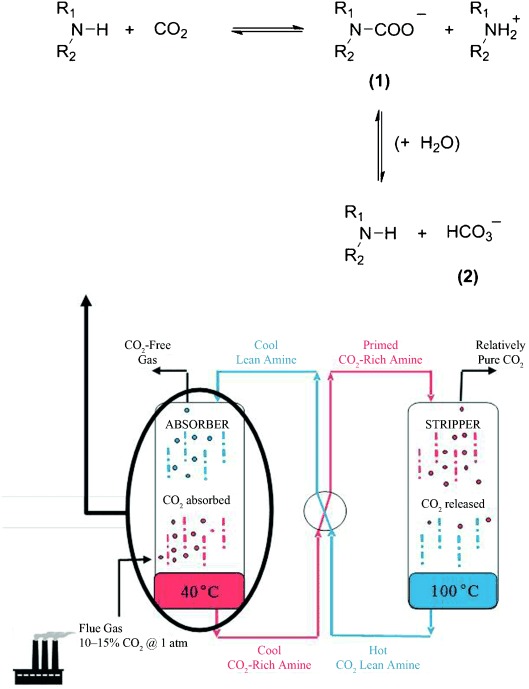
Reaction mechanism leading to carbamate (**1**) formation for the reaction of CO_2_ with primary (R_1_R_2_NH, where R_1_ or R_2_=H) and secondary (R_1_R_2_NH) amines (top),^[^^10^^]^ which occurs in the absorption step of the PCC process (bottom).

The CO_2_ absorption/desorption by aqueous amine-based absorbents has been, and continues to be, extensively studied in the search for improvements in PCC efficiency. The CO_2_ absorption/desorption process is shown schematically in Figure 1. Typically, CO_2_ reacts with aqueous amines to generate carbamate (**1**), bicarbonate (**2**) and a protonated amine. The amine substitution pattern affects the products produced. Primary amines such as MEA and secondary amines like diethanolamine (DEA) and piperazine (PZ) react with CO_2_ under aqueous conditions to form a carbamate derivative R_1_R_2_NCOO^−^ (Figure 1). The 2:1 reaction stoichiometry restricts CO_2_ absorption capacity of primary and secondary amines to a theoretical upper limit of 0.5 mol CO_2_/mol amine. However, it has been demonstrated that the carbamate can be hydrolysed at high CO_2_ loadings (>0.5 mol CO_2_/mol amine) to produce bicarbonate and regenerate a free amine,^10^ which allows for slightly improved absorption capacities (Figure 1). Given the chemical stability of carbamates formed from 1° and 2° amines, hydrolysis does not occur at an industrially relevant rate,^11^ with the exception of heterocyclic secondary amines such as piperidine, which have been demonstrated to form a labile carbamate that is readily hydrolysed.^[^^10^^]^

Tertiary amines (R_1_R_2_R_3_N) cannot react directly with CO_2_ to form carbamates.^[^^12^–^14^^]^ Tertiary amines are believed to act as catalysts facilitating the hydrolysis reaction between CO_2_ and OH^−^ to form bicarbonate.^[^^12^^,^ ^14^^,^ ^15^^]^ This 3°-amine pathway is kinetically and thermodynamically less favourable than carbamate formation.^[^^16^^]^ Bicarbonate formation is advantageous in consuming only one molecule of amine per molecule of CO_2_, allowing for increased CO_2_ absorption capacities.

CO_2_ absorption by aqueous amines is a reversible process, and the degree of reversibility is amine-dependent. Amines that form stable carbamates exhibit faster reaction rates, but a larger input of energy is required for absorbent regeneration. Conversely, amines that form more bicarbonate than carbamate exhibit slower reaction rates and require less energy for regeneration. Recent technological advances have allowed for the convenient and rapid analysis of these chemical species to be carried out in situ during the PCC absorption/desorption cycle by attenuated total reflectance Fourier transform infrared (ATR-FTIR) spectroscopy. In particular, the ability of ATR-FTIR to distinguish carbamate from bicarbonate formation accelerates the screening of potential PCC amines.^[^^10^^,^ ^17^^]^

**Figure 2:**
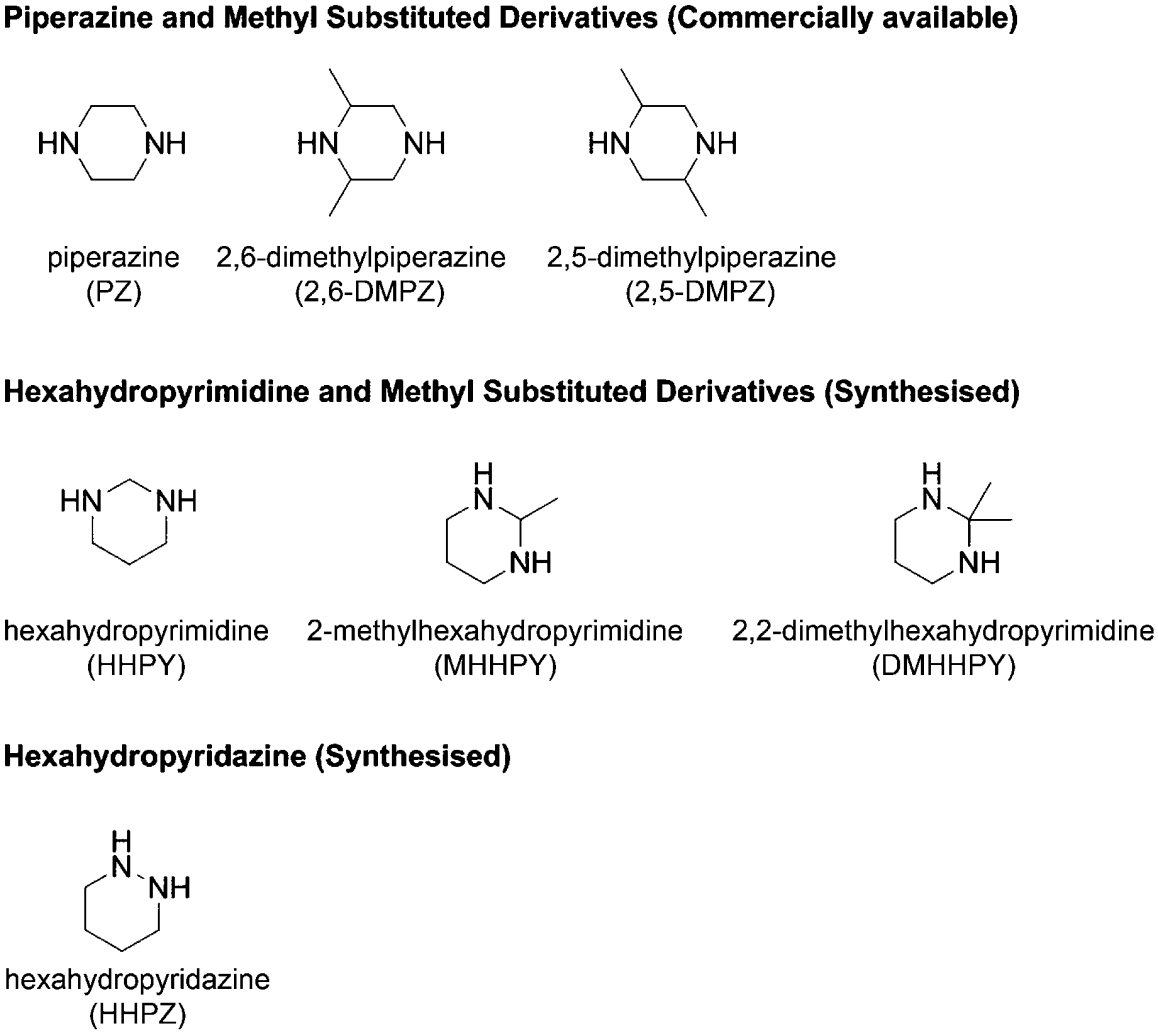
Chemical structures of the heterocyclic amines examined for CO_2_ absorption properties.

We recently reported the application of ATR-FTIR in a model PCC absorbent system with substituted piperidines.^[^^10^^]^ Herein we report on the in situ CO_2_ absorption characteristics of a series of heterocyclic diamines (Figure 2): piperazine (PZ), 2,6-dimethyl- and 2,5-dimethylpiperazine (2,6-DMPZ and 2,5-DMPZ), hexahydropyrimidine (HHPY), 2-methylhexahydropyrimidine (MHHPY), 2,2-dimethylhexahydropyrimidine (DMHHPY) and hexahydropyridazine (HHPZ).

## 2. Results and Discussion

### 2.1. Infrared Spectral Analysis

The effect of structural variations on the CO_2_ absorption characteristics of the heterocyclic diamines shown in Figure 2 were assessed in relation to the IR-identifiable products, that is, carbamate versus bicarbonate absorbance; CO_2_ absorption capacity, defined as moles of CO_2_ absorbed per mole of amine in solution (mol CO_2_/mol amine); and the initial rate of CO_2_ absorption (mol CO_2_/mol amine min^−1^). Full details of our experimental approach is given in the Experimental Section and also in our previous work.^[^^10^^]^ Each experiment was conducted until equilibrium was established and a maximum CO_2_ loading achieved. This was amine-dependent but typically required 45–90 mins. Calculations were also performed to investigate the electronic/steric effects of the structural variations on the amine-carbamate derivatives.

#### 2.1.1. Piperazine (PZ)

**Figure 3:**
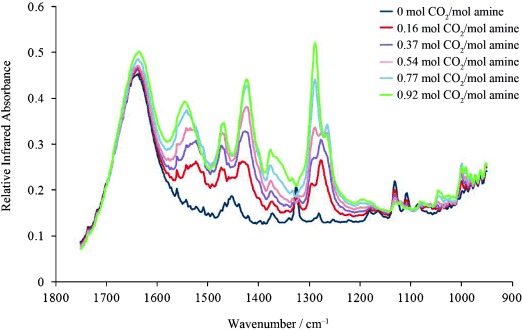
Partial IR spectral profile of an aqueous solution of PZ (1.5 mol L^−1^) as CO_2_ is absorbed to a maximum loading of 0.92 mol CO_2_/mol amine.

Our investigations commenced with parent heterocyclic diamine PZ (Figure 2). As can be seen from the partial (1750–950 cm^−1^) FTIR spectrum collected during a typical CO_2_ absorption experiment with an aqueous PZ solution (1.5 mol L^−1^), five major FTIR peaks evolve during CO_2_ absorption (Figure 3). The carbamate (NCOO^−^) derivatives of heterocyclic monoamines have been identified as giving rise to several strong absorbance bands in the 1600–1260 cm^−1^ region, including the asymmetric 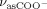, 1600–1500 cm^−1^) and symmetric 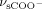, 1450–1350 cm^−1^) vibrations of the COO^−^ moiety and the N–COO^−^ stretching vibration 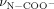, 1300–1260 cm^−1^) of the NCOO^−^ derivative.^[^^10^^]^ The protonated amine (NH_2_^+^) generated on absorption of CO_2_ was found to give rise to an absorbance band in the 1479–1474 cm^−1^ region (NH_2_^+^ bending mode). In an amine/CO_2_/H_2_O system, the bicarbonate (HCO_3_^−^) species was identified as giving rise to a broad peak in the 1360–1354 cm^−1^ region (ν_sC–O_).^[^^10^^,^ ^17^^]^ Assignment was based on the spectral data acquired for 1-methylpiperidine (tertiary amine)/CO_2_/H_2_O and 2-amino-2-methyl-1-propanol (AMP)/CO_2_/H_2_O systems. It is known that the absorption of CO_2_ by aqueous AMP (α-dimethyl-substituted MEA derivative) leads to the formation of mostly HCO_3_^−^ with no significant NCOO^−^ formation.^[^^14^^,^ ^18^^,^ ^19^^]^ Herein these peaks can be related to the vibrational modes of the potential ionic reaction products, including PZ-carbamate (PZ-COO^−^), protonated PZ (PZ-H^+^) and bicarbonate (HCO_3_^−^). PZ, being a secondary diamine, should react with CO_2_ in solution to form NCOO^−^, predominately in the form of a protonated PZ-COO^−^ derivative (^+^H_2_NR_1_R_2_NCOO^−^).^[^^20^^,^ ^21^^]^ One amine moiety acts as the absorption site for CO_2_, and the other as a proton acceptor. PZ has also been reported to form the dicarbamate (^−^OOC-PZ-COO^−^), which was detected by ^1^H and ^13^C NMR spectroscopy, at CO_2_ loadings of 0.2–0.8 mol CO_2_/mol amine.^[^^20^^,^ ^21^^]^

**Figure 4:**
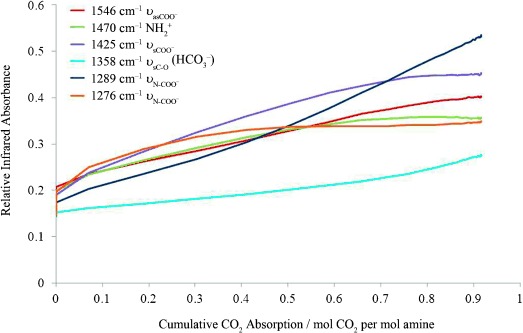
Relationship between cumulative CO_2_ absorption of an aqueous solution of PZ (1.5 mol L^−1^) and IR absorbance for the bands assigned to the vibrational modes of NCOO^−^ and HCO_3_^−^.

The FTIR spectra for the PZ/CO_2_/H_2_O system closely resembles that we previously reported for the piperidine/CO_2_/H_2_O system, differing only in slight shifts in key IR stretching frequencies.^10^ At low levels of absorbed CO_2_ the PZ/CO_2_/H_2_O system exhibits the 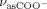 (1524 cm^−1^), 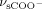 (1432 cm^−1^) and 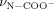 (1276 cm^−1^ and 1294 cm^−1^) of the PZ-COO^−^ derivative and the NH_2_^+^ vibration of PZ-H^+^ (1470 cm^−1^). These peaks shift to 1546, 1425 and 1289 cm^−1^, respectively, with increasing CO_2_ absorption levels (Figure 3). As anticipated, a near-linear relationship between cumulative CO_2_ absorption and IR peak intensity is observed for the spectral peaks assigned to 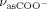, 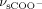 and PZ-H^+^. Increased peak absorbance is concomitant with the rate of NCOO^−^ formation at the reaction onset plateauing as a maximum CO_2_ loading of 0.92 mol CO_2_/mol amine is approached (Figure 4). This near-linear relationship differs from that observed for the 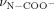 bands at 1276 and 1294 cm^−1^. From the data presented in Figures 3 and 4 the primary 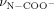 absorbance emerged at 1276 cm^−1^ and was the dominant peak, but only at CO_2_/mol amine loadings of 0.4–0.5 mol CO_2_/mol amine. At amine loadings greater than 0.5 CO_2_/mol amine the 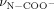 absorbance decreases correspondingly with a sharp increase in intensity of the absorbance band at 1294 cm^−1^ and a frequency shift to 1289 cm^−1^. This trend in 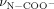 peak absorbance in the 1294–1276 cm^−1^ region is attributed to formation of ^−^OOC-PZ-COO^−^. The IR absorbance of PZ/CO_2_/H_2_O in this region differs from those of all other heterocyclic amine and diamine systems thus far reported, and the remaining subset of secondary heterocyclic diamines analysed in this study (see below) displays only a single 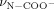 absorbance band in the 1283–1272 cm^−1^ region.

As the IR stretching frequencies of PZ-dicarbamate had not been previously reported we turned to computational approaches to facilitate the assignment of key vibrational modes of PZ-COO^−^, in particular 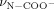. Calculations were performed at the B3LYP/6-31+G** and MP2/6-31+G** levels (gas phase, Spartan ‘08).^[^^22^^]^

The B3LYP/6-31+G** calculations assigned PZ-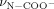 to a single band at 1282 cm^−1^ (no scaling), while MP2/6-31+G** positioned this band at 1284 cm^−1^ (no scaling), similar in shape, but not as broad, as that which initially emerges at 1276 cm^−1^ in Figure 3. For the ^−^OOC-PZ-COO^−^ species B3LYP/6-31+G** gave two sharp 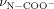 absorbances at 1297–1266 and 1348–1345 cm^−1^, which correlated well with MP2/6-31+G** calculated positions of 1302–1274 cm^−1^ and 1364–1355 cm^−1^. These values correlate well with the experimentally observed peaks at values of 1266, 1276 and 1294 cm^−1^, with the latter two shifting to 1289 cm^−1^ with CO_2_ absorption. The B3LYP/6-31+G** and MP2/6-31+G** calculations confirm our peak assignments for the PZ-CO_2_ carbamate absorption species above.

The evolution of a weak broad absorbance band in the 1360–1350 cm^−1^ region of the PZ/CO_2_/H_2_O IR spectral profile (Figure 3) was assigned to ν_sC–O_ of HCO_3_^−^. This absorbance band was far less prominent than that we observed for the piperidine/CO_2_/H_2_O system. Additionally, this absorbance in the PZ system does not follow the trend observed with the corresponding piperidine system; that is, the depletion of 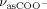, 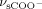 and 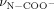 absorbance bands with concomitant increase in the HCO_3_^−^ absorbance band for CO_2_ loadings greater than 0.5 mol CO_2_/mol amine.^[^^10^^]^ The increase in HCO_3_^−^ absorbance in the piperidine system is attributable to hydrolysis of the initially formed carbamate, which strongly suggests, consistent with the IR data presented herein, that the PZ system forms a hydrolysis-resistant carbamate.

#### 2.1.2. 2,6- and 2,5- Dimethyl-Substituted Piperazine Derivatives (2,6-DMPZ and 2,5-DMPZ)

**Figure 5:**
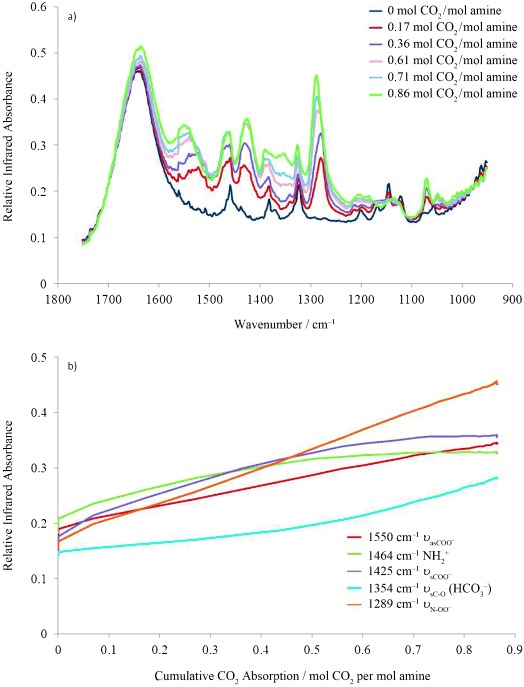
a) Partial IR spectral profile collected for an aqueous solution of 2,6-DMPZ (1.5 mol L^−1^) as CO_2_ is absorbed to a maximum loading of 0.86 mol CO_2_/mol amine. b) Relationship between the cumulative CO_2_ absorption and IR absorbance for 2,6-DMPZ.

**Figure 6:**
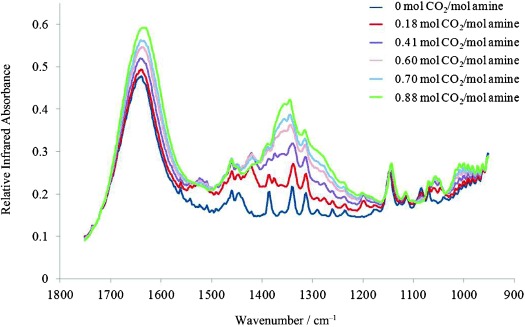
Partial IR spectral profile of an aqueous solution of 2,5-DMPZ (1.5 mol L^−1^) as CO_2_ is absorbed to a maximum loading of 0.88 moles CO_2_ per mole of amine.

The effect of alkyl substituents on the CO_2_ absorption characteristics of PZ was examined with 2,6-dimethylpiperazine (2,6-DMPZ) and 2,5-dimethylpiperazine (2,5-DMPZ) (Figure 2). The IR spectral profiles obtained for 2,6-DMPZ/CO_2_/H_2_O and 2,5-DMPZ/CO_2_/H_2_O are shown in Figures 5 and 6, respectively.

The IR spectral data collected for the 2,6-DMPZ/CO_2_/H_2_O system (Figure 5 a and b) are almost identical to those recorded for the equivalent PZ system. While the 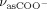 and 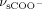 bands were less intense and only a single 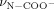 absorbance band evolves in the 1289–1276 cm^−1^ region, all major absorbances of the 2,6-DMPZ system are within 6 cm^−1^ of those of the PZ system: 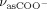 at 1526 cm^−1^; 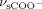 at 1425 cm^−1^; 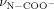 at 1279 cm^−1^ for 2,6-DMPZ-COO^−^; NH_2_^+^ bending of 2,6-DMPZ-H^+^ at 1464 cm^−1^; and HCO_3_^−^ absorbance at 1354 cm^−1^. The 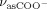 and 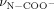 absorbances shift to 1550 and 1289 cm^−1^, respectively, with increasing CO_2_ absorption. The lack of a second 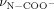 absorbance is reflected in the relationship between absorbance and cumulative CO_2_ absorption (Figure 5).

Due to steric congestion arising from the two α-CH_3_ moieties, initial CO_2_ absorption most likely occurred at the less hindered and more nucleophilic amine moiety, resulting in NCOO^−^ formation. This reduced nucleophilicity and hence reactivity towards CO_2_ hindered dicarbamate formation, correlating with the observation of a single 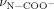 peak in the IR spectrum. The reduced prevalence of dicarbamate formation resulted in increased hydrolysis and HCO_3_^−^, as evidenced by rapid growth of the ν_sC–O_ band at 1354 cm^−1^ (Figure 5 a, b). The α-dimethylamine moiety acted catalytically, in a manner analogous to that reported for sterically hindered amines, to accelerate formation of HCO_3_^−^.^[^^10^^]^

The subtle structural variations between 2,6-DMPZ and 2,5-DMPZ resulted in a significant change in the IR profile. In the case of the 2,5-DMPZ/CO_2_/H_2_O system the most dominant feature is HCO_3_^−^ absorbance, as evidenced by the intense peak in the 1400–1300 cm^−1^ region. There is little evidence to support formation of a stable carbamate (Figure 6).^[^^10^^,^ ^17^^]^

#### 2.1.3. Hexahydropyrimidine (HHPY)

**Figure 7:**
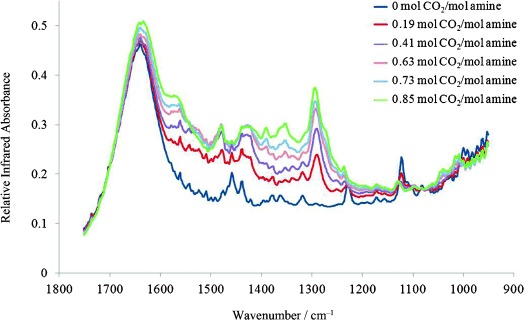
Partial IR spectral profile collected for an aqueous solution of HHPY (1.5 mol L^−1^) as CO_2_ is absorbed to a maximum loading of 0.85 mol CO_2_/mol amine.

The HHPY/CO_2_/H_2_O system displayed some similarity with the PZ/CO_2_/H_2_O system in terms of signal positioning but with evidently weaker signals, due in part to the lower concentration of amine (HHPY) available for this study. Notwithstanding this, Figure 7 shows evolution of 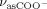 at 1570–1520 cm^−1^, 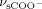 at 1427 cm^−1^ and 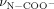 at 1293 cm^−1^ of HHPY-COO^−^; the NH_2_^+^ bending mode of HHPY-H^+^ at 1479 cm^−1^; and HCO_3_^−^ absorbance at 1354 cm^−1^. The HCO_3_^−^ absorbance band was more prominent than that observed for the PZ/CO_2_/H_2_O system, that is, HHPY forms a more labile NCOO^−^ derivative that is more susceptible to hydrolysis.

#### 2.1.4. 2-Methylhexahydropyrimidine (MHHPY)

**Figure 8:**
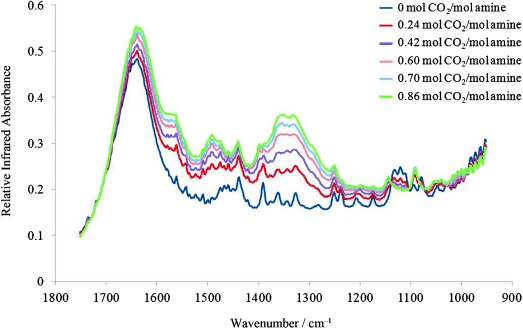
Partial IR spectral profile collected for an aqueous solution of MHHPY (1.5 mol L^−1^) as CO_2_ is absorbed to a maximum loading of 0.86 mol CO_2_/mol amine.

The IR spectrum of the MHHPY/CO_2_/H_2_O system is dominated by the broad HCO_3_^−^ absorbance band in the 1400–1300 cm^−1^ region (Figure 8), which is characteristic of α-substituted amines such as AMP.^[^^10^^,^ ^17^^]^ MHHPY is the methyl-substituted analogue of HHPY. Given the intensity of the HCO_3_^−^ band in Figure 8, MHHPY was readily hydrolysed under the study conditions, with HCO_3_^−^ formation dominating on absorption of CO_2_.

#### 2.1.5. 2,2-Dimethylhexahydropyrimidine (DMHHPY)

**Figure 9:**
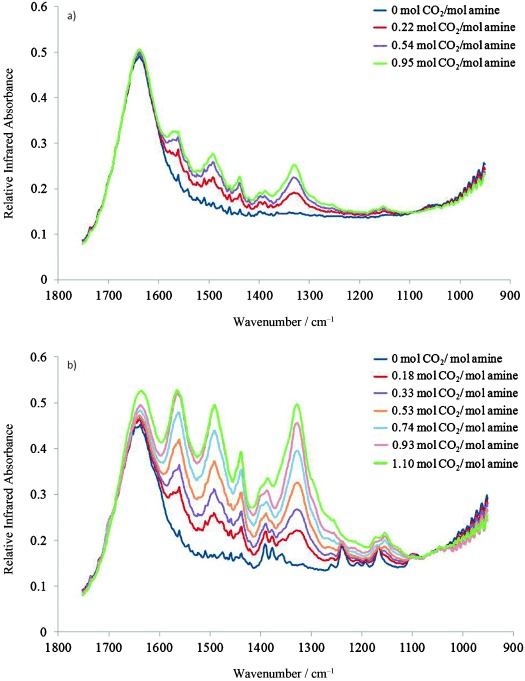
Partial IR spectral profile of an aqueous solution of a) synthesised DMHHPY (1.5 mol L^−1^) and b) DAP (0.6 mol L^−1^) as CO_2_ is absorbed to a maximum loading of 1.10 and 0.95 mol CO_2_/mol amine, respectively.

Given the structural similarity between DMHHPY and MHHPY, we anticipated predominant HCO_3_^−^ formation on CO_2_ absorption by DMHHPY. However the IR profile obtained for the DMHHPY/CO_2_/H_2_O system (Figure 9 a) was significantly different to that obtained with MHHPY (Figure 8) and the HHPY and PZ systems (Figures 7 and 3, respectively). Here DMHHPY is acting more in keeping with a blended-amine PPC absorbent system. Close examination of the in-house synthesized DMHHPY revealed the presence of unconverted 1,3-diaminopropane (DAP) which had been unavoidably carried forward to the final product. Hence, the contamination of DMHHPY with DAP explains the observed blended-system-like IR profile (Figure 9 a).

Re-examination of the IR spectrum of the DMHHPY/CO_2_/H_2_O system identified the NH bending mode of 1,3-diaminopopane at 1602 cm^−1^. While 1,3-diaminopropane [approximately 37 %, 1.91 g (^1^H NMR)] was the minor component within the DMHHPY (63 %, 3.24 g)/CO_2_/H_2_O system, it formed predominately the corresponding carbamate (DAP) on CO_2_ absorption.

To allow potential deconvolution of the DAP and DMHHPY signals in the original DMHHPY/CO_2_/H_2_O IR profile, data were collected separately for a DAP/CO_2_/H_2_O system at a DAP concentration of 0.6 mol L^−1^ (Figure 9 b). It is apparent that the original DMHHPY system is dominated by the reactivity of DAP (cf. Figure 9 a, b). Both systems show evolution of 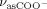 (1565 and 1568 cm^−1^, respectively), 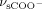 (1440 cm^−1^) and 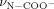 (1328 and 1330 cm^−1^, respectively) of the DAP-COO^−^ derivative and the NH_3_^+^ bending mode of protonated DAP (1492 cm^−1^). For the blended DMHHPY/CO_2_/H_2_O system, weaker absorbance bands were also observed to emerge at 1385 and 1370–1350 cm^−1^ at CO_2_ loadings above 1.0 mol CO_2_/mol amine. These new peaks are consistent with NCOO^−^ hydrolysis and HCO_3_^−^ formation. Carbamate hydrolysis was not observed for the pure DAP/CO_2_/H_2_O system.

**Figure 10:**
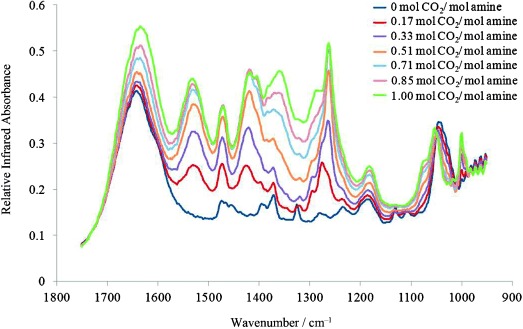
Partial IR spectral profile of an aqueous solution of an AMP/PZ blend (2.4/0.6 mol L^−1^, respectively) as CO_2_ is absorbed to a maximum loading of 1.00 mol CO_2_/mol amine.

**Figure 11:**
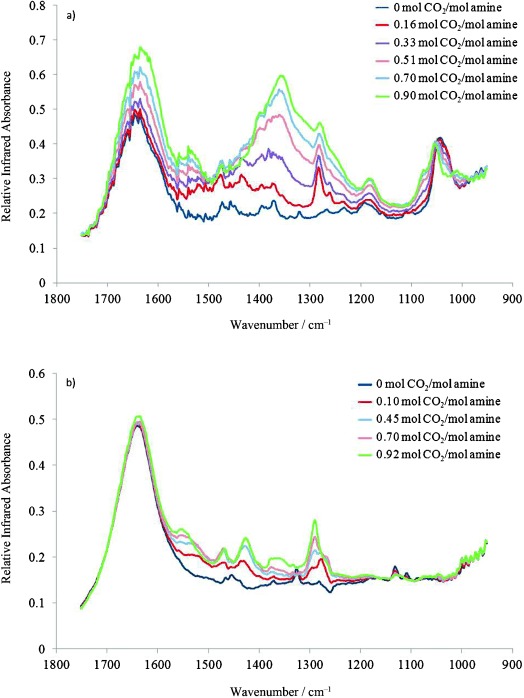
Partial IR spectral profile of an aqueous solution of a) unblended AMP (3 mol L^−1^) and b) unblended PZ (0.6 mol L^−1^) as CO_2_ is absorbed to a maximum loading of 0.84 and 0.92 mol CO_2_/mol amine, respectively.

In the initial DMHHPY/CO_2_/H_2_O system (Figure 9 a) the DAP-COO^−^ absorbance bands dominate the IR spectrum. However in the DAP/CO_2_/H_2_O systems the carbamate absorbances are considerably weaker, the initial effect of which was thought to be that the DAP concentration on the DMHHPY system appears to be significantly higher than the 0.6 mol L^−1^ evident in Figure 9 b. However, a similar difference in intensity between the carbamate absorbance bands of a blended AMP (2.4 mol L^−1^)/PZ (0.6 mol L^−1^) system (Figure 10, further described below) versus an unblended PZ (0.6 mol L^−1^) system was also observed (Figure 11). Carbamate absorbance in the unblended PZ system was found to be considerably weaker than that observed for the AMP/PZ blended system, despite equivalent PZ concentrations (6 mol L^−1^). Based on the percentage concentrations determined by ^1^H NMR spectroscopy the ratio of DMHHPY to DAP in the blended system was 0.95/0.85 mol L^−1^ (total concentration 1.8 mol L^−1^).

For comparative purposes an AMP/PZ blended amine system (2.4 mol L^−1^/0.6 mol L^−1^, respectively) was also investigated. Similarly to the blended DMHHPY absorbent, the AMP/PZ blend consists of an amine that forms predominately HCO_3_^−^ on absorption of CO_2_ (AMP, major constituent) and an amine that forms predominately NCOO^−^ (PZ, minor constituent). A similar trend in IR absorbance was observed in the spectral data collected for the AMP/PZ/CO_2_/H_2_O system (Figure 10) to that described above for the blended DMHHPY/CO_2_/H_2_O system. Despite PZ being the minor constituent of the amine blend, the PZ-carbamate absorbance bands dominated the IR spectral profile. Figure 10 shows the evolution of ν_ascoo_- (1533 cm^−1^), ν_scoo_- (1421 cm^−1^) and 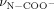 (1276 cm^−1^ and shifts to 1263 cm^−1^) of the PZ-COO^−^ derivative and the NH_2_^+^ vibration of PZ-H^+^ (1471 cm^−1^). HCO_3_^−^ absorbance was seen to emerge in the 1386–1330 cm^−1^ region after a CO_2_ loading of about 0.5 mol CO_2_/mol amine. For comparison Figure 11 a and b present the IR spectral profile for an unblended AMP/CO_2_/H_2_O system (3 mol L^−1^) and PZ/CO_2_/H_2_O system (0.6 mol L^−1^), respectively. The HCO_3_^−^ absorbance band was more prominent in the IR spectra of the AMP/PZ/CO_2_/H_2_O system compared to that of the DMHHPY/CO_2_/H_2_O system. This was most likely due to the difference in amine concentrations, with the AMP/PZ blend having a total concentration of 3 mol L^−1^ and the DMHHPY/1,3-diaminopropane blend a total concentration of 1.5–1.8 mol L^−1^.

#### 2.1.6. Hexahydropyridazine

HHPZ absorbed no CO_2_ during a typical CO_2_ absorption/FTIR experiment. HHPZ is a hydrazine derivative that is reported to have a p*K*_a_ value of 7.9,^[^^23^^]^ which is much lower than that of PZ (9.73),^[^^24^^]^ HHPY (9.75)^[^^25^^]^ or 2,5-DMPZ (9.66).^[^^26^^]^ The low basicity of HHPZ compared to the other diamines (p*K*_a_>9.5) would significantly reduce the reactivity of the amine towards CO_2_.

### 2.2. Absorption Capacity and Absorption Rate

Having established the ability of our diamines to absorb CO_2_, the initial absorption rate *R*_IA_ and absorption capacity *C*_A_ were determined. The *R*_IA_ value was measured by a thermal gravimetric analysis (TGA) method, and *C*_A_ was measured simultaneously with the IR spectral data (see Experimental Section). These data are presented in Table 1. For comparison, the reactivity of conventional absorbents MEA, DEA and AMP (α-dimethyl-substituted MEA) are also included.

**Table 1:** Measured absorption capacity *C*_A_ at 40 °C for an amine concentration of 1.5 mol L^−1^ and initial absorption rate *R*_IA_ at 40 °C and an amine concentration of 1.5 mol L^−1^ for aqueous solutions of PZ, 2,6-DMPZ, 2,5-DMPZ and synthesised amines HHPY, MHHPY, DMHHY and HHPZ. For comparison, the reactivity of conventional absorbents such as MEA, DEA and AMP (1.5 mol L^−1^, unless otherwise stated) are included.

Amine	*C*_A_^[a]^	*R*_IA_^[b]^
PZ	0.92	0.045
2,6-DMPZ	0.86^[c]^	0.025^[d]^
2,5-DMPZ	0.88	0.018^[e]^
HHPY	0.85	0.032
MHHPY	0.86	0.018
DMHHPY	1.33	0.032
HHPZ	0	0
MEA	0.56^[12]^, ^[f]^	0.027
DEA	0.60^[12]^, ^[f]^	0.015
AMP	0.84^[12]^, ^[f]^	0.006

[a] Mol CO_2_/mol amine; data used to calculate *C*_A_ were measured in the absorption reactor/FTIR system.

[b] Mol CO_2_/mol amine, min^−1^; data used to calculate *R*_IA_ were measured by microscale TGA. Initial absorption rates were calculated by using linear regression to determine the slope of the absorption capacity curve. *R*^2^≥0.995.

[c] A precipitate formed during CO_2_ absorption/FTIR.

[d] A precipitate formed during the CO_2_ and N_2_ runs of the TGA experiment.

[e] A precipitate formed during the CO_2_ run of the TGA experiment. This could be mainly due to the evaporation of water.

[f] 3 mol L^−1^ concentration analysed.

The current industry-standard PCC amine MEA returned *C*_A_=0.56 mol CO_2_/mol amine and *R*_IA_=0.027 mol CO_2_/mol amine min^−1^. From the data amassed for PZ, 2,6-DMPZ, HHPY and DMHHPY in Table 1, superior *C*_A_ and *R*_IA_ values were observed for all these diamines relative to MEA. Superior *C*_A_ values were also observed for 2,5-DMPZ and MHHPY, but with lower *R*_IA_ values. HHPZ did not react with CO_2_, and DMHHPY was blended with DAP. Diamine *C*_A_ values ranged from 0.85 (HHPY) to 0.92 (PZ) mol CO_2_/mol amine and *R*_IA_ values from 0.018 (2,5-DMPZ) to 0.045 (PZ) mol CO_2_/mol amine min^−1^. While in absolute terms PZ was the standout pure diamine with the highest *C*_A_ (0.92 mol CO_2_/mol amine) and *R*_IA_ (0.045 mol CO_2_/mol amine min^−1^), *C*_A_ and *R*_IA_ are not the sole factors to be considered in determining the most efficient PCC diamine absorbent; aqueous solubility and stability of the carbamate also play a role. HPPY displays higher water solubility than PZ, on the basis of observations when preparing 1.5 mol L^−1^ amine solutions. HHPY was readily soluble at this concentration, as opposed to PZ, which required heating and stirring for dissolution. Additionally, HHPY displays high *C*_A_ (0.85 mol CO_2_/mol amine) and *R*_IA_ (0.032 mol CO_2_/mol amine min^−1^; Table 1) and showed clear evidence of formation of a hydrolysis-susceptible carbamate (see above and Figure 7).

The PZ analogues 2,6-DMPZ and 2,5-DMPZ displayed lower *R*_IA_ values of 0.025 and 0.018 mol CO_2_/mol amine min^−1^, respectively. As these analogues differ only in the number of methyl substituents (PZ has none) and their positioning, these data suggested that introduction of methyl moieties had an adverse effect on the initial rate of CO_2_ absorption. 2,6-DMPZ has both methyl groups α to a single NH group, while 2,5-DMPZ has one methyl group α to each NH group. The measured *R*_IA_ values indicate that the effect of addition of α-methyl groups is cumulative, with *R*_IA_ dropping from 0.032 (PZ) to 0.025 (2,6-DMPZ) to 0.018 mol CO_2_/mol amine min^−1^ (2,5-DMPZ). Concurrent with the reduction in *R*_IA_ was an increased prevalence towards HCO_3_^−^ formation for 2,5-DMPZ (see above and Figure 6). The reactivity of 2,6-DMPZ towards CO_2_ was found to be similar to that of MEA, with the exception of a higher *C*_A_ value. The propensity of 2,5-DMPZ for HCO_3_^−^ formation was similar to that observed with MHHPY (see above and Figure 8), which was reflected in the almost identical *C*_A_ (0.88 and 0.86 mol CO_2_/mol amine respectively) and *R*_IA_ (0.018 and 0.018 mol CO_2_/mol amine min^−1^, respectively) values obtained for these amines. The reactivity of 2,6-DMPZ towards CO_2_ was found to be similar to that of MEA, with the exception of a higher *C*_A_ value. 2,6-DMPZ was found to form predominantly carbamate on absorption of CO_2_ (Figure 5), similar to HHPY (Figure 7). While the propensity for carbamate hydrolysis and subsequent HCO_3_^−^ formation is also similar to that observed with HHPY (cf. Figures 5 and 7), which was reflected in the almost identical *C*_A_ (0.88 and 0.85 mol CO_2_/mol amine, respectively) values, 2,6-DMPZ returned a lower *R*_IA_ value (0.025 and 0.032 mol CO_2_/mol amine min^−1^, respectively). Despite forming predominately HCO_3_^−^, the initial absorption rates obtained for both MHHPY and 2,5-DMPZ were much higher than that obtained for AMP and comparable to those of MEA and DEA (Table 1).

Of the diamines examined, DMHHPY exhibited the highest *C*_A_ (1.1 mol CO_2_/mol amine) value and an *R*_IA_ value higher than that of MHHPY and comparable to that of HHPY. This is an artefact of the serendipitous blending with DAP, which contributes significantly to the observed CO_2_ absorption capacity. Aqueous DAP has *C*_A_=0.95 mol CO_2_/mol amine. The bicarbonate-forming DMHHPY further promotes CO_2_ absorption, resulting in *C*_A_>1.0 mol CO_2_/mol amine. The 1° amino groups of DAP contribute towards DMHHPY’s increased initial reaction rate compared to MHHPY.

### 2.3. Effect of Structural Variations on Carbamate Structures

The effect of diamine structural variation on the ability to form stable carbamates was examined at the B3LYP/6-31+G** and MP2/6-31+G** levels of theory. Geometry optimisations were initially performed on PZ, 2,6-DMPZ, 2,5-DMPZ, HHPY, MHHPY, DMHHPY and HHPZ. Table 2 lists selected atomic properties including electrostatic potential (ESP) partial charges on the amino nitrogen atoms and the exposed area on the nitrogen atoms for these diamines. The trends in results obtained at the two levels of theory were found to be in good agreement with one another.

**Table 2:** ESP charges and exposed areas on the nitrogen atoms [Å^2^] for optimised forms of the diamines analysed herein.

Amine		ESP charge on N		Exposed area on N [Å^2^]	
		B3LYP	MP2	B3LYP	MP2
PZ	N1	−0.584	−0.640	4.71	4.80
	N2	−0.584	−0.640	4.71	4.80
2,6-DMPZ	N1	−0.718	−0.752	4.21	4.25
	N2	−0.752	−0.778	4.70	4.82
2,5-DMPZ	N1	−0.746	−0.785	4.45	4.54
	N2	−0.746	−0.785	4.45	4.54
HHPY	N1	−0.874	−0.907	4.91	4.96
	N2	−0.874	−0.907	4.91	4.96
MHHPY	N1	−0.851	−0.881	4.77	4.83
	N2	-0.728	-0.759	4.57	4.65
DMHHPY	N1	−0.959	−0.979	4.50	4.57
	N2	−0.957	−0.982	4.51	4.57
HHPZ	N1	−0.525	−0.551	6.82	6.89
	N2	−0.386	−0.402	5.71	5.77

**Figure 12:**
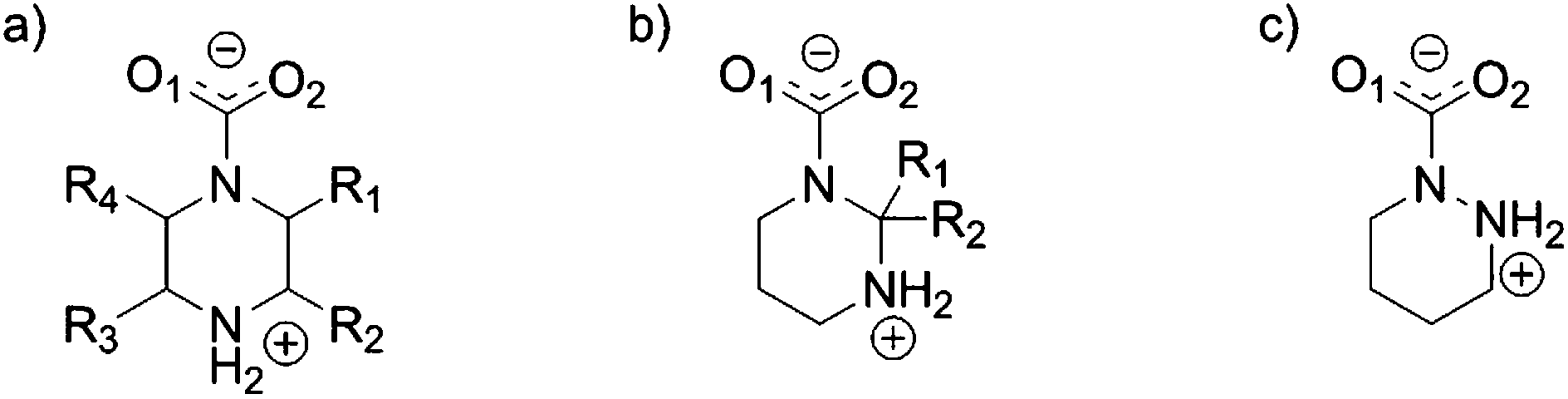
Chemical structural associated with Table 3 with regards to *r*_N–C,_ *r*_C1–O1_/*r*_C1–O2_. a) 2,6-DMPZ (1. R_2_=R_3_=CH_3_, R_1_=R_4_=H and 2. R_1_=R_4_=CH_3_, R_2_=R_3_=H) and 2,5-DMPZ (R_2_=R_4_=CH_3_, R_1_=R_3_=H). b) HHPY (R_1_=R_2_=H), MHHPY (R_1_=CH_3_, R_2_=H) and DMHHPY (R_1_=R_2_=CH_3_). c) HHPZ.

Diamines can react with CO_2_ in aqueous solution to form three possible forms of carbamate species: amine carbamate (HNR_1_R_2_NCOO^−^), protonated amine carbamate (^+^H-HNR_1_R_2_NCOO^−^) and dicarbamate. Of these three forms, ^+^HHNR_1_R_2_NCOO^−^ was expected to be the main reaction product, with one amino group acting as the binding site for CO_2_ and the other as a proton acceptor. Geometry optimisations were next performed for the protonated amine-carbamate (^+^H-HNR_1_R_2_NCOO^−^) and amine-carbamate (HNR_1_R_2_NCOO^−^) species. For the lowest-energy conformer of each ^+^HHNR_1_R_2_NCOO^−^ and HNR_1_R_2_NCOO^−^ derivative, Table 3 provides the calculated N–COO^−^ bond length (*r*_N–C_ [Å]), *r*_C1–O1_/*r*_C1–O2_ [Å] (Figure 12) and ESP partial negative charges on both oxygen atoms as a measure of charge delocalisation.

**Table 3:** Calculated N–COO^−^ bond lengths *r*_N–C_ [Å], *r*_C1–O1_/*r*_C1–O2_ [Å] and ESP partial charge on both oxygen atoms for optimised geometries of the ^+^HHNR_1_R_2_NCOO^−^ and HNR_1_R_2_NCOO^−^ derivatives of the subset of diamines analysed.

Carbamate derivative	*r*_N−C_ [Å]		*r*_C–O1_/*r*_C–O2_ [Å]		ESP charge on O1/O2	
	B3LYP	MP2	B3LYP	MP2	B3LYP	MP2
H^+^-PZ-carbamate	1.513	1.507	1.247/1.247	1.256/1.256	−0.690/−0.690	−0.681/−0.681
PZ-carbamate	1.471	1.477	1.257/1.257	1.264/1.264	−0.743/−0.743	−0.739/−0.739
H^+^-2,6-DMPZ-carbamate^[a]^	1.508	1.505	1.248/1.249	1.256/1.256	−0.688/−0.689	−0.680/−0.680
2,6-DMPZ-carbamate^[a]^	1.469	1.477	1.258/1.258	1.264/1.264	−0.772/−0.772	−0.767/−0.767
H^+^-2,6-DMPZ-carbamate^[b]^	1.523	1.527	1.247/1.247	1.255/1.255	−0.691/−0.692	−0.699/−0.698
2,6-DMPZ-carbamate^[b]^	1.461	1.463	1.260/1.260	1.266/1.266	−0.753/−0.753	−0.749/−0.749
H^+^-2,5-DMPZ-carbamate^[c]^	1.505	1.503	1.249/1.247	1.259/1.253	−0.699/−0.665	−0.702/−0.656
2,5-DMPZ-carbamate^[c]^	1.465	1.470	1.259/1.258	1.266/1.264	−0.750/−7.35	−0.746/−0.731
H^+^-HHPY-carbamate	1.507	1.503	1.227/1.277^[d]^	1.235/1.284^[d]^	−0.641/−0.735^[d]^	−0.625/−0.736^[d]^
HHPY-carbamate	1.475	1.478	1.257/1.259	1.263/1.267	−0.762/−0.767	−0.744/−0.751
H^+^- MHHPY-carbamate	1.417	1.417	1.211/1.354^[d]^	1.211/1.354^[d]^	−0.650/−0.772^[d]^	−0.640/−0.782^[d]^
MHHPY-carbamate	1.463	1.469	1.258/1.260	1.264/1.266	−0.753/−0.770	−0.746/−0.759
H^+^-DMHHPY-carbamate	1.555	1.550	1.235/1.244	1.243/1.253	−0.653/−0.699	−0.648/−0.702
DMHHPY-carbamate	1.501	1.500	1.252/1.261	1.258/1.268	−0.758/−0.819	−0.743/−0.816
H^+^-HHPZ-carbamate	1.390	1.396	1.215/1.350^[d]^	1.221/1.353^[d]^	−0.575/−0.660^[d]^	−0.566/−0.670^[d]^
HHPZ-carbamate	1.460	1.470	1.254/1.263	1.260/1.267	−0.741/−0.759	−0.736/−0.758

[a] Isomer 1: R_2_=R_3_=CH_3_ and R_1_=R_4_=H (Figure 12).

[b] Isomer 2: R_1_=R_4_=CH_3_ and R_2_=R_3_=H (Figure 12).

[c] R_2_=R_4_=CH_3_ and R_1_=R_3_=H (Figure 12).

[d] Hydrogen bonding between O2 of the carbamate moiety and a proton of the NH_2_^+^ group (Figure 12).

As anticipated, methyl substitution significantly increased the partial negative charges on the amino groups of 2,6-DMPZ (N1 −0.718; N2 −0.752), 2,5-DMPZ (N1, N2 −0.746), MHHPY (N1 −0.851; N2 −0.728) and DMHHPY (N1 −0.959; N2 −0.957) relative to PZ (N1, N2 −0.584) and reduced the exposed area on the amino nitrogen atom of 2,6-DMPZ (N1 4.21 Å^2^; N2 4.70 Å^2^), 2,5-DMPZ (N1, N2 4.45 Å^2^), MHHPY (N1 4.77 Å^2^; N2 4.47 Å^2^) and DMHHPY (N1 4.50 Å^2^; N2 4.51 Å^2^) relative to PZ (N1, N2 4.71 Å^2^) at the B3LYP level. These changes in electronic effects both have impact on the resonance structure of the carboxylate moiety and hence overall stability of the carbamate derivative (see below).

Studies herein (Figure 3 and 4) demonstrated that PZ forms a hydrolysis-resistant NCOO^−^ derivative, and thus the optimised geometries of H^+^-PZ-COO^−^ and PZ-COO^−^ were used as the baseline against which the remaining amine NCOO^−^ derivatives were compared. For both H^+^-PZ-COO^−^ and PZ-COO^−^ resonance stabilisation of the carboxylate moiety is evident, with calculations showing identical charges on O1 and O2 as well as displaying identical *r*_C–O_ values. The *r*_C–O_ values of 1.247 and 1.257 Å reveal partial double-bond character for H^+^-PZ-COO^−^ and PZ-COO^−^, respectively (Table 3). The N–COO^−^ bond length of both PZ-COO^−^ species was of single-bond character [standard single-bond *r*_N–C_ in PZ is 1.466 Å (B3LYP) and 1.465 Å (MP2)]. H^+^-2,6-DMPZ-COO^−^ and 2,6-DMPZ-COO^−^ also exhibit this stable resonance structure, allbeit with slightly shorter N–COO^−^ bond lengths. These findings are in keeping with our IR studies on 2,6-DMPZ and PZ (Figure 5), which gave very similar outcomes, with the exception of the emergence of a small HCO_3_^−^ absorbance band. The protonated and unprotonated 2,6-DMPZ-COO^−^ forms of isomer 2 (Figure 12), the minor reaction component, contributed to HCO_3_^−^ formation.

H^+^-HHPY-COO^−^ displays lower levels of charge delocalisation across the two oxygen atoms. Changes in charge distribution and bond length were noted with *r*_C–O1_=1.227 Å and *r*_C–O2_=1.277 Å, which were mirrored in the change in electron density at O1 (−0.641) and O2 (−0.735) (B3LYP). The shift in electron distribution was much less pronounced in the HHPY-COO^−^ species with an *r*_C–O1_ of 1.257 Å and a *r*_C–O2_ of 1.259 Å which was mirrored in the change in electron density at O1 (−0.762) and O2 (−0.767) (B3LYP). The N–COO^−^ bond lengths of H^+^-HHPY-COO^−^ (1.507 Å) and HHPY-COO^−^ (1.475 Å) are similar to that of the PZ-COO^−^ species (1.471 Å). These findings are in keeping with our IR studies (Figure 7), in which HHPY was identified as forming a more labile NCOO^−^ derivative that is more susceptible to hydrolysis than that of PZ. The lowest energy conformer obtained for H^+^-HHPY-COO^−^, as opposed to HHPY-COO^−^ species, exhibited intramolecular hydrogen bonding between the COO^−^ group and the NH_2_^+^ moiety. The low-energy conformers of H^+^-MHHPY-COO^−^ and H^+^-HHPZ-COO^−^ were also found to exhibit the same intramolecular hydrogen bonding. The resonance structure of the H^+^-MHHPY-COO^−^ was less delocalised with *r*_C–O1_=1.211 Å and *r*_C−O2_=1.354 Å, which was mirrored in the changes in electron density at O1 (−0.650) and O2 (−0.772) (B3LYP). The N–COO^−^ bond length of H^+^-MHHPY-COO^−^ was found to be much shorter (1.417 Å). The shift in electron distribution of the carboxylate resonance structure was again much less pronounced in the MHHPY-COO^−^ species compared to the H^+^-MHHPY-COO^−^ species. MHHPY-COO^−^ has a shorter N–COO^−^ bond length (1.463 Å) than PZ-COO^−^ (1.471 Å) and HHPY-COO^−^ (1.475 Å). MHHPY forms predominantly HCO_3_^−^ on absorption of CO_2_, as does 2,5-DMPZ. Both species display shorter N–COO^−^ bonds and lower levels of resonance stabilisation.

In our IR studies DMHHPY was found to be representative of a blended amine system. Nonetheless, optimised geometries of the H^+^-DMHHPY-COO^−^ and DMHHPY-COO^−^ were still analysed, and both exhibited reduced resonance in the carboxylate moiety with *r*_C–O1_=1.235 Å and *r*_C–O2_=1.244 Å, which was mirrored in the changes in electron density at O1 (−0.653) and O2 (−0.699), and with *r*_C–O1_=1.252 Å and *r*_C–O2_=1.261 Å, which was mirrored in the changes in electron density at O1 (−0.758) and O2 (−0.819) (B3LYP), respectively. Given the structural similarity of DMHHPY and MHHPY, DMHHPY was expected to form predominantly HCO_3_^−^ on absorption of CO_2_.

Experimentally HHPZ was unreactive towards CO_2_. Calculations revealed H^+^-HHPZ-COO^−^ to have a significantly shorter N–COO^−^ bond length of 1.390 Å (significant double-bond character), as well as the largest displacement in electron distribution of the carboxylate resonance structure with *r*_C−O1_=1.215 Å and *r*_C–O2_=1.350 Å, which was mirrored in the changes in electron density at O1 (−0.575) and O2 (−0.660) (B3LYP). This was much less pronounced in the HHPZ-COO^−^ species; nonetheless, in a diamine system it is typical for one amino group to act as binding site for CO_2_ while the other is protonated. These data support our experimental observations.

## 3. Conclusions

A series of heterocyclic diamines (PZ, 2,6-DMPZ, 2,5-DMPZ, HHPY, MHHPY, DMHHPY and HHPZ) have been evaluated as potential PCC absorbents by in situ ATR-FTIR spectroscopy. Of these diamines, PZ displayed both the highest CO_2_ absorption capacity (*C*_A_=0.92 mol CO_2_/mol amine) and highest initial absorption rate (*R*_IA_=0.045 mol CO_2_/mol amine min^−1^). These values represent a significant enhancement over currently used amines such as MEA. PZ forms a hydrolysis-resistant carbamate, as well as a dicarbamate. This behaviour is unique to PZ. Hydrolysis of the carbamate derivative of HHPY was observable in the IR spectra collected during CO_2_ absorption. HHPY displayed similar CO_2_ absorption characteristics to PZ, but with a higher propensity for HCO_3_^−^ formation. The introduction of α-methyl substituents increased the propensity towards carbamate hydrolysis and HCO_3_^−^ formation. Additionally α-methyl substitution decreased *R*_IA_, with PZ analogues 2,6-DMPZ and 2,5-DMPZ displaying lower *R*_IA_ values of 0.025 and 0.018 mol CO_2_/mol amine min^−1^, respectively. Increasing the number of methyl groups α to the NH group also increases the rate of HCO_3_^−^ formation. Despite forming predominately HCO_3_^−^, the *R*_IA_ of MHHPY (0.018 mol CO_2_/mol amine min^−1^) was much higher than that of the corresponding α-dimethyl substituted 1° amine AMP (*R*_IA_=0.006 mol CO_2_/mol amine min^−1^) and comparable with that of the industrially relevant MEA and DEA. The serendipitously blended DAP/DMHHPY exhibited the highest *C*_A_ (1.1 mol CO_2_/mol amine) and excellent *R*_IA_ (0.032 mol CO_2_/mol amine min^−1^). HHPZ was found to be relatively unreactive towards CO_2_. In all instances our calculations at the B3LYP/6-31+G** and MP2/6-31+G** levels of theory supported our experimental observation. Finally, we propose that HHPY offers the best compromise between high CO_2_ absorption capacity, carbamate formation, hydrolysis to HCO_3_^−^ and water solubility for future use in model, and potentially pilot-scale, PCC systems.

## Experimental Section

General: All starting materials were purchased from Sigma Aldrich and used without further purification. Solvents were bulk and distilled prior to use. ^1^H and ^13^C NMR spectra were recorded on a Bruker Avance AMX 300 MHz spectrometer at 300.1315 and 75.4762 MHz, respectively. Chemical shifts *δ* are reported relative to internal standards. Mass spectra were recorded on a Shimadzu LCMS-2010 EV spectrometer and obtained by the ESI method. IR spectra were recorded by ATR-FTIR on a Mettler-Toledo ic10 FTIR: spectrometer.

Preparation of Hexahydropyrimidine (HHPY): Formaldehyde (37 wt % solution, 1.5 mol, 126 g) was added dropwise to an ice-cooled, stirred aliquot of anhydrous 1,3-diaminopropane (1 mol, 74.1 g) over 30 mins. The reaction mixture was then stirred for 24 h at room temperature and cooled in an ice bath, and NaOH was added (1 mol, 40 g). The organic layer was transferred to a Dean–Stark apparatus and azeotropically distilled with cyclohexane (100 mL). The cyclohexane was removed in vacuo and the residue fractionally distilled to afford anhydrous hexahydropyrimidine (17 g, 20 %) as a clear oil (b.p. 57–60 °C/27 mbar, lit. ^[^^25^^,^ ^27^^]^: b.p. 58–60 °C/20 mm Hg). ^1^H NMR (300 MHz, CDCl_3_): *δ*=1.36 (2 H, m), 1.96 (2 H, br s), 2.82 (4 H, t, *J*=5.5 Hz), 3.63 ppm (2 H, s); ^13^C NMR (75 MHz, CDCl_3_): *δ*=28.61, 45.65, 62.79 ppm; MS (ESI+): *m*/*z* 87 (*M*+1); FTIR: ν_NH_ 3267 cm^−1^.

Preparation of 2-Methylhexahydropyrimidine (MHHPY): Acetaldehyde (0.215 mol, 9.5 g) in diethyl ether (100 mL) was added dropwise to ice-cooled 1, 3-diaminopropane (0.2 mol, 14.8 g). The reaction mixture was then stirred over K_2_CO_3_ (0.4 mol, 55.4 g) for 24 h at room temperature. The solvent was removed in vacuo and the residue fractionally distilled to afford 2-methylhexahydropyrimidine (17.5 g, 87 %) as a clear oil (b.p. 56–59 °C/30 mbar, lit. ^[^^28^^]^ b.p. 60 °C/30 mm Hg). ^1^H NMR (300 MHz, CDCl_3_) *δ*=0.80 (3 H, d, *J*=6.0 Hz), 1.09 (2 H, m), 2.48 (2 H, m), 2.76 (4 H, m), 3.25 ppm (1 H, q, *J*=6.0 Hz); ^13^C NMR (75 MHz, CDCl_3_) *δ*=22.75, 26.80, 45.41, 67.09 ppm; MS (ESI+: *m*/*z* 101 [*M*+1]; FTIR: ν_NH_ 3266 cm^−1^.

Preparation of 2, 2-Dimethylhexahydropyrimidine (DMHHPY): A solution of acetone (0.215 mol, 12.5 g) in diethyl ether (100 mL) was added dropwise to ice-cooled 1,3-diaminopropane (0.2 mol, 14.83 g). The reaction mixture was then stirred over K_2_CO_3_ (0.4 mol, 55.4 g) for 24 h at room temperature. The solvent was removed in vacuo and the residue fractionally distilled to afford 2, 2-dimethylhexahydropyrimidine (16.17 g, 71 %) as a clear oil (b.p. 50–54 °C/25 mbar, lit. ^[^^28^^]^: b.p. 65 °C/25 mm Hg). ^1^H NMR (300 MHz, CDCl_3_): *δ*=0.90 (6 H, s), 1.05 (2 H, m), 1.45 (2 H, s), 2.60 ppm (4 H, t, *J*=5.7 Hz); ^13^C NMR (75 MHz, CDCl_3_): *δ*=27.26, 36.87, 39.99, 64.31 ppm; MS (ESI+): *m*/*z* 115 [*M*+1]; FTIR: ν_NH_ 3271, 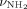 1602 cm^−1^.

Preparation of Hexahydropyridazine (HHPZ): Dibromobutane (0.1 mol, 21.6 g) was added dropwise to a stirred solution of diethylhydrazine dicarboxylate (0.1 mol, 17.6 g), K_2_CO_3_ (0.2 mol, 27.7 g) and acetonitrile (100 mL) at room temperature. The reaction mixture was then heated to reflux for 24 h, cooled and filtered. The solvent was removed in vacuo and the residue fractionally distilled to afford 1, 2-dicarbethoxyhexahydropyridazine (18.5 g, 80 %) as a clear oil (b.p. 110–114 °C/2–3 mbar, lit. ^[^^29^^]^: 106–114 °C/3 mm Hg). ^1^H NMR (300 MHz, CDCl_3_): *δ*=1.07 (6 H, m), 1.49 (4 H, s), 2.74 (4 H, br s), 3.97 ppm (4 H, m); ^13^C NMR (75 MHz, CDCl_3_): *δ*=14.36, 23.19, 44.76, 61.93, 155.15 ppm; MS (ESI+): *m*/*z* 231 [*M*+1]; FTIR: ν_C=O_ 1706 cm^−1^.

1, 2-Dicarbethoxyhexahydropyridazine (0.13 mol, 30.1 g) was added to a solution of NaOH (0.72 mol, 20.8 g), water (20 mL) and methanol (150 mL). The reaction mixture was then heated to reflux for 20 h, cooled and the precipitated inorganic salt removed by filtration. The filtrate was heated to reflux for a further 20 h, cooled, filtered and the solvent removed in vacuo. The residue was extracted with dichloromethane (40 mL×3), concentrated in vacuo and fractionally distilled to afford hexahydropyridazine (6.75 g, 60 %) as a yellow oil (b.p. 38–40 °C/11 mbar, lit. ^[^^30^^]^: b.p. 38 °C/8 mm Hg). ^1^H NMR (300 MHz, MeOD): *δ*=2.39 (4 H, m), 3.73 (4 H, m), 5.72 ppm (2 H, s); ^13^C NMR (75 MHz, MeOD): *δ*=23.06, 47.00 ppm; MS (ESI+): *m*/*z* 87 [*M*+1]; FTIR: ν_NH_ 3269 cm^−1^.

Microscale Thermogravimetric Analysis (TGA): A Setaram Labsys TG-DTA/DSC thermogravimetric analyser (TGA) was used in isothermal mode at 40 °C to analyse aqueous CO_2_/amine reactivity on a microscale (100 μL). 1.5 mol L^−1^ aqueous amine solutions were exposed to a gas stream of 15 vol % CO_2_ (>99.9 % purity, BOC Australia) in N_2_ at atmospheric pressure. A gas flow rate of 30 mL min^−1^ was used for all experiments.

To determine total CO_2_ uptake two separate TGA experiments were performed for each amine. The first experimental run determined the mass loss due to evaporation when the test solution was exposed to a 100 % N_2_ gas stream. The second experimental run determined the mass increase of the test solution when exposed to CO_2_ (15 vol % CO_2_ in N_2_ gas stream) over the same length of time. Each experiment was performed on a fresh 100 μL aliquot of the test solution in a 100 μL alumina crucible (Setaram). From the data collected an absorption curve was then calculated for each amine by subtracting the mass at time *t* of the evaporation run from the mass at time *t* of the absorption run. Initial absorption rates could then be calculated by using linear regression to determine the slope of the absorption curve.

Absorption/FTIR Experiments: The absorption reactor apparatus used to analyse aqueous CO_2_/amine reactivity has been described previously.^[^^10^^]^ Briefly, a gas stream of 13 vol % CO_2_ (>99.9 % purity, BOC Australia) in N_2_ with a flow rate of 1.8 L min^−1^ was bubbled through a 1.5 mol L^−1^ aqueous amine solution in a glass reactor vessel, maintained at 40 °C by a temperature-controlled water bath (Techne). The CO_2_ content of both the gas inflow and outflow was measured by using a Horiba VA 3000 CO_2_ analyser. The difference between the CO_2_ concentration of the reactor gas inflow and gas outflow was used determine the amount of CO_2_ absorbed by the amine solution (mol CO_2_/mol amine). Each experiment was run until the measured CO_2_ concentration in the outflow returned to the original percentage value, that is, equilibrium was reached. A typical run lasted between 45 and 90 mins. Solution volumes of 30 mL were used for all experiments.

For the duration of each absorption experiment an ATR diamond-tipped IR probe, coupled via a mirrored K6 conduit to an ic10 FTIR spectrometer (Mettler-Toledo), was immersed in the aqueous amine solution. In situ IR measurements were obtained simultaneously with the CO_2_ absorption measurements, with the FTIR spectrometer set to continuously collect spectra for the duration of the absorption experiment over the spectral range of 4000–650 cm^−1^. Each spectrum was recorded as the average of 256 scans over a sampling interval of fifteen seconds with a resolution of 4 cm^−1^. The amines investigated were synthesised according to the procedures detailed above, with the exception of commercially available PZ, 2,6-DMPZ and 2,5-DMPZ (Sigma-Aldrich).

Computational Details: The computational software package Spartan ‘08 was used to calculate and compare optimised geometries (gas phase) of the heterocyclic diamines and their carbamate derivatives.^[^^22^^]^ First, molecular mechanics calculations using the MMFF94 force field and Monte Carlo search algorithm were used to obtain a set of low-energy conformers for each amine and carbamate molecule. Each subset of low-energy conformers were then re-submitted as a geometry optimisation at the B3LYP/6-31+G** and MP2/6-31+G** levels to obtain an equilibrium geometry corresponding to an energy minimum (characterized by a gradient <0.001). Vibrational analysis was performed for all optimised geometries to ensure that they correspond to local minima, that is, there are no imaginary frequencies.
